# Kidney-differentiated cells derived from Lowe Syndrome patient’s iPSCs show ciliogenesis defects and Six2 retention at the Golgi complex

**DOI:** 10.1371/journal.pone.0192635

**Published:** 2018-02-14

**Authors:** Wen-Chieh Hsieh, Swetha Ramadesikan, Donna Fekete, Ruben Claudio Aguilar

**Affiliations:** 1 Department of Biological Sciences, Purdue University, West Lafayette, IN United States of America; 2 Purdue Institute for Integrative Neuroscience, Purdue University, West Lafayette, IN United States of America; 3 Purdue University Center for Cancer Research, Purdue University, West Lafayette, IN United States of America; 4 Purdue Institute for Inflammation, Immunology and Infectious Disease, Purdue University, West Lafayette, IN United States of America; University of Texas at Austin Dell Medical School, UNITED STATES

## Abstract

Lowe syndrome is an X-linked condition characterized by congenital cataracts, neurological abnormalities and kidney malfunction. This lethal disease is caused by mutations in the *OCRL1* gene, which encodes for the phosphatidylinositol 5-phosphatase Ocrl1. While in the past decade we witnessed substantial progress in the identification and characterization of LS patient cellular phenotypes, many of these studies have been performed in knocked-down cell lines or patient’s cells from accessible cell types such as skin fibroblasts, and not from the organs affected. This is partially due to the limited accessibility of patient cells from eyes, brain and kidneys. Here we report the preparation of induced pluripotent stem cells (iPSCs) from patient skin fibroblasts and their reprogramming into kidney cells. These reprogrammed kidney cells displayed primary cilia assembly defects similar to those described previously in cell lines. Additionally, the transcription factor and cap mesenchyme marker Six2 was substantially retained in the Golgi complex and the functional nuclear-localized fraction was reduced. These results were confirmed using different batches of differentiated cells from different iPSC colonies and by the use of the human proximal tubule kidney cell line HK2. Indeed, *OCRL1* KO led to both ciliogenesis defects and Six2 retention in the Golgi complex. In agreement with Six2’s role in the suppression of ductal kidney lineages, cells from this pedigree were over-represented among patient kidney-reprogrammed cells. We speculate that this diminished efficacy to produce cap mesenchyme cells would cause LS patients to have difficulties in replenishing senescent or damaged cells derived from this lineage, particularly proximal tubule cells, leading to pathological scenarios such as tubular atrophy.

## Introduction

The Oculo-Cerebro-Renal syndrome of Lowe (OCRL), also known as Lowe syndrome (LS) is a genetic disease caused by mutations in the *OCRL1* gene which encodes for an inositol 5-phosphatase (EC 3.1.3.36) [1]. This X-linked condition is characterized by bilateral cataracts at birth, mental retardation and kidney malfunction, with the latter being the most common cause of death of affected children [1,2]. Specifically, patients display tubulopathy and Fanconi-like syndrome that often evolves into kidney failure [1,3,4]. However, how these clinical manifestations develop is still poorly understood.

Nevertheless, a series of cellular phenotypes have been reported in Ocrl1 deficient cells, notably abnormalities in the assembly of the so-called primary cilia (PC) [5–8]. This axoneme-based structure constitutes a specialization of the plasma membrane enriched in receptors and channels that plays a crucial role in signal transduction [9–12]. PC signaling activities are particularly relevant during embryonic development, but they are also crucial for adult cell function. Abnormalities in PC assembly or function invariably lead to a broad group of developmental diseases collectively known as ciliopathies [13–15]. Given the broad functional relevance of the PC, these pathological conditions are characterized by multi-organ compromise, including brain/eye/kidney abnormalities [13,14,16]. These observations further highlight the potential relevance of PC phenotypes as an underlying cause of LS symptoms. However, the existence of PC abnormalities have not been investigated in kidney cells of LS patients. One obstacle to achieve this goal has been the limited availability of patient cells from affected organs. Although some kidney cells can be isolated and expanded from urine samples, unpredictable yields and the need of repeating such a laborious process with each patient (to capture patient variability in terms of *OCRL1* mutations and genetic background/modifiers) represents a challenge. In addition, this approach is not suitable for brain- and eye-derived cell types.

Here we report the successful generation of induced Pluripotent Stem Cells (iPSCs) from biopsy-accessible skin fibroblasts. Reprogramming of such iPSCs into cell types that are difficult or cumbersome to obtain from patients allows mechanistic investigations of cellular phenotypes. Considering their relevance for LS patient health status and ultimate outcome, we reprogrammed iPSCs as kidney cells and monitored their ability to assemble PC. We found that LS kidney-reprogrammed cells had significantly less ability than normal to undergo successful ciliogenesis. Further, we also found that these LS kidney-reprogrammed Six2-positive cells showed a substantial decrease of proper nuclear localization of the transcription factor in comparison to normal controls. Surprisingly, LS kidney cells increasingly retained Six2 in the Golgi complex. As expected considering Six2 inhibitory effects on ductal differentiation, Cytokeratin-8 (Ck8)-positive ductal lineage cells were over-represented among LS kidney cells.

As a whole, this work established a suitable strategy to obtain different cell types from LS patients, while maintaining their particular genetic background characteristics. Importantly, this study also verified the existence of PC assembly abnormalities in patient-derived LS kidney cells, suggesting a role for this phenotype in the renal pathology. Finally, we also showed that LS cells yielded disproportionate populations of ductal versus cap mesenchyme lineages. Cap mesenchymal-derived cells produce important lineages such as tubular cells (known to be affected in LS patients), leading us to speculate that this abnormality may have consequences on the ability of affected individuals to replenish their tubular cells upon injury/insult or wearing of cells.

## Results

A variety of cellular phenotypes associated with Ocrl1-deficiency have been described, including phosphatidylinositol (4, 5) bisphosphate [PI(4, 5)P_2_] accumulation [17,18], actin and RhoGTPase regulation abnormalities [17,19–23], trafficking [17,24–27] and ciliogenesis abnormalities [5–8]. However, most of these phenotypes have been observed under Ocrl1 knock-down conditions and/or in LS patient skin fibroblasts.

Since LS is characterized by a marked organ-specificity (mainly affecting brain, eyes and kidneys), it is conceivable that different cell types may vary in phenotypic penetrance of specific abnormalities (such as cilia assembly defects). Therefore, to assess phenotype disease relevance it is necessary to ascertain whether cells from LS-affected organs display similar cellular abnormalities as those described in patient fibroblasts. Further, different patient mutations and genetic backgrounds may affect phenotype penetrance. These factors underscore the need for suitable approaches able to yield organ-specific cells from multiple patients.

Since the availability of cells from patients’ affected organs (*e*.*g*., brain, eyes and kidneys) is limited while skin fibroblasts are readily accessible, we used the latter cells to carry out a kidney reprogramming strategy. Further, patient-specific, cell replacement therapies are promising approaches for many diseases that can take advantage of such developments [28].

Given the relevance of renal function for the patient well-being/outcome, here we focused on fibroblast reprogramming into kidney cells and on testing the presence of primary cilia phenotypes (known to affect kidney function—[29–32]. In addition, this strategy allowed us to also investigate whether Ocrl1-deficiency affected the ability to produce renal progenitors and/or to maintain a replenishing pool of cells in the kidney.

### Kidney-reprogrammed cells show ciliogenesis defects

#### Induced Pluripotent Stem Cell (iPSC) generation from LS patient and normal fibroblasts

Since it has been shown that Ocrl1-deficient cells display primary cilia (PC) defects [5–8], and this phenotype is expected to lead to kidney-related abnormalities, we proceeded to monitor ciliogenesis in kidney-reprogrammed cells. As a first step towards the obtention of kidney cells from patient fibroblasts we prepared iPSC to reprogram using standard protocols.

**Fig 1:**
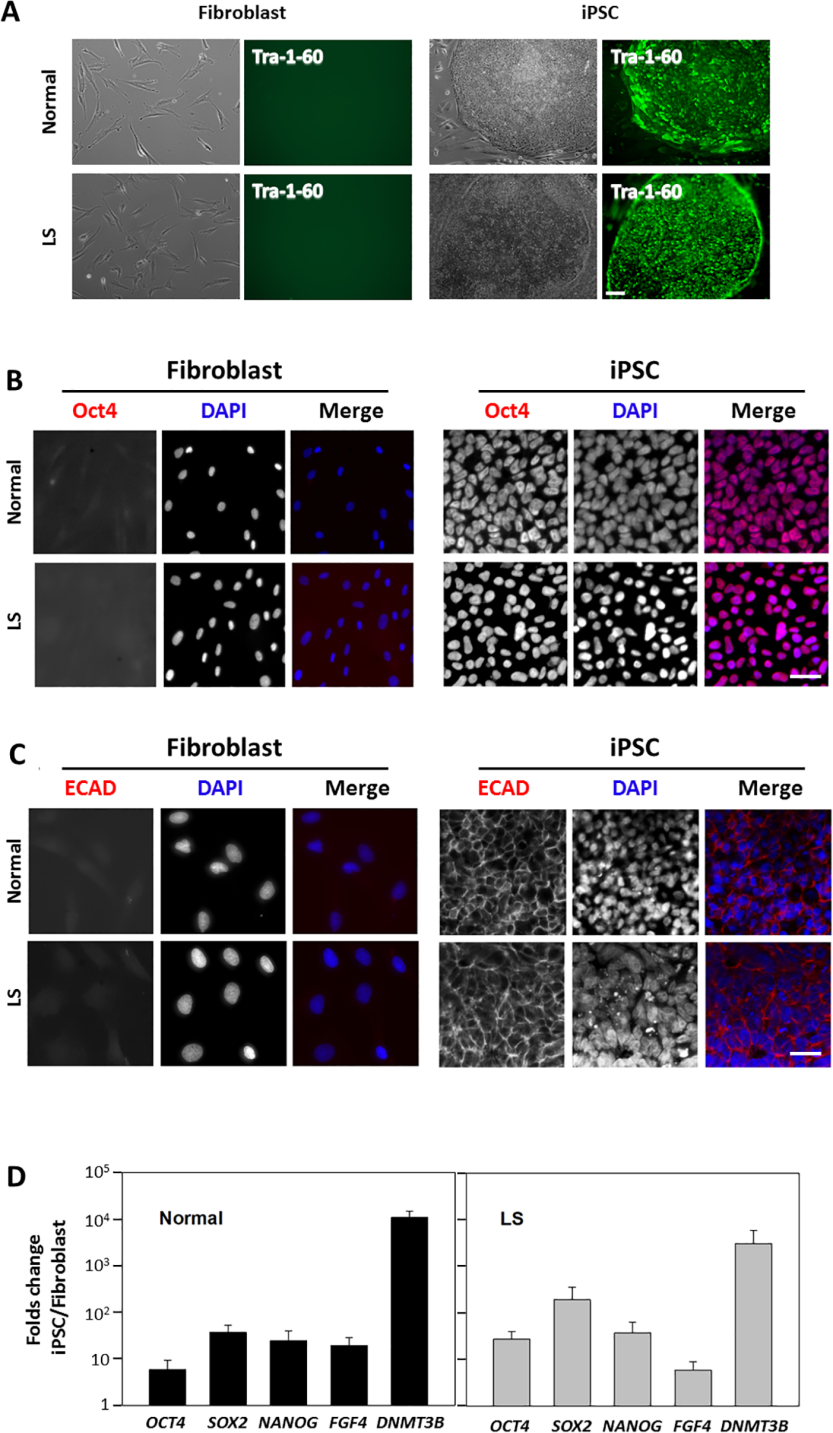
Generation of normal- and LS patient-derived iPSCs from skin fibroblasts. **A.** iPSCs from normal and patient fibroblasts were prepared as described in Materials and methods. Cell and colony morphology differences were evident using bright-field microscopy, expression of the iPSC marker Tra-1-60 was investigated using direct immunofluorescence of live fibroblasts and iPSCs (see text for details). Scale bar: 100μm. **B** and **C**. Expression and localization of the transcription factor Oct4 (**B**) and adhesion molecule E-cadherin (ECAD, **C**) was investigated in normal/LS patient fibroblasts (*left*) and iPSCs (*right*) using indirect immunofluorescence with specific antibodies (red). DAPI staining (blue) was performed to highlight nucleus position. Scale bar: 10μm. **D**. Expression of several stem cell markers was investigated by quantitative RT-PCR as described in Materials and methods. Results were expressed as folds change observed in iPSC with respect to the corresponding fibroblast, results were also controlled with respect to RPLP0 expression. A representative experiment is shown.

Normal and LS skin fibroblasts were transfected with a retroviral Yamanaka’s cocktail [33] for expression of *OCT4*, *SOX2*, *KLF4* and *c-MYC* as described in Materials and methods. After approximately 30–35 days, colonies arose in which cells displayed evidence of substantial morphological change, yielding iPSC-typical cuboidal, small cells, packed in tight groups (Fig 1A). Importantly, some of these colonies showed robust expression of the stem cell marker Tra-1-60 (Fig 1A). Such colonies were isolated and expanded for further characterization. As expected, these cells also expressed the Yamanaka’s cocktail transfected proteins: *e*.*g*., the transcription factor Oct4 (Fig 1B), which represses epithelial-to-mesenchyme transition [34,35]. Importantly, candidate colonies also showed expression of the Klf4-downstream target E-cadherin which favors colony cohesion, thus reinforcing an epitheloid phenotype (Fig 1C). In addition to the presence of iPSC-characteristic Tra-1-60 and E-cadherin (both absent in skin fibroblasts—see Fig 1A and 1C), we investigated the expression of other stemness markers by quantitative RT-PCR (Fig 1D). Specifically, we demonstrated an increase of more than 5-fold in the qPCR signal associated with different stem cell-markers as compared to their corresponding source fibroblasts (Fig 1D). Since karyotypic abnormalities have been shown to occur in hESs and hiPSCs [36], we also verified the presence of normal karyotypes for both normal and LS iPSCs (data not shown).

Based on the characterization described above, we isolated, expanded and preserved iPSC colonies. No major differences were detected between normal and LS iPSCs in passage frequency and morphology.

#### Kidney-lineage differentiation

**Fig 2:**
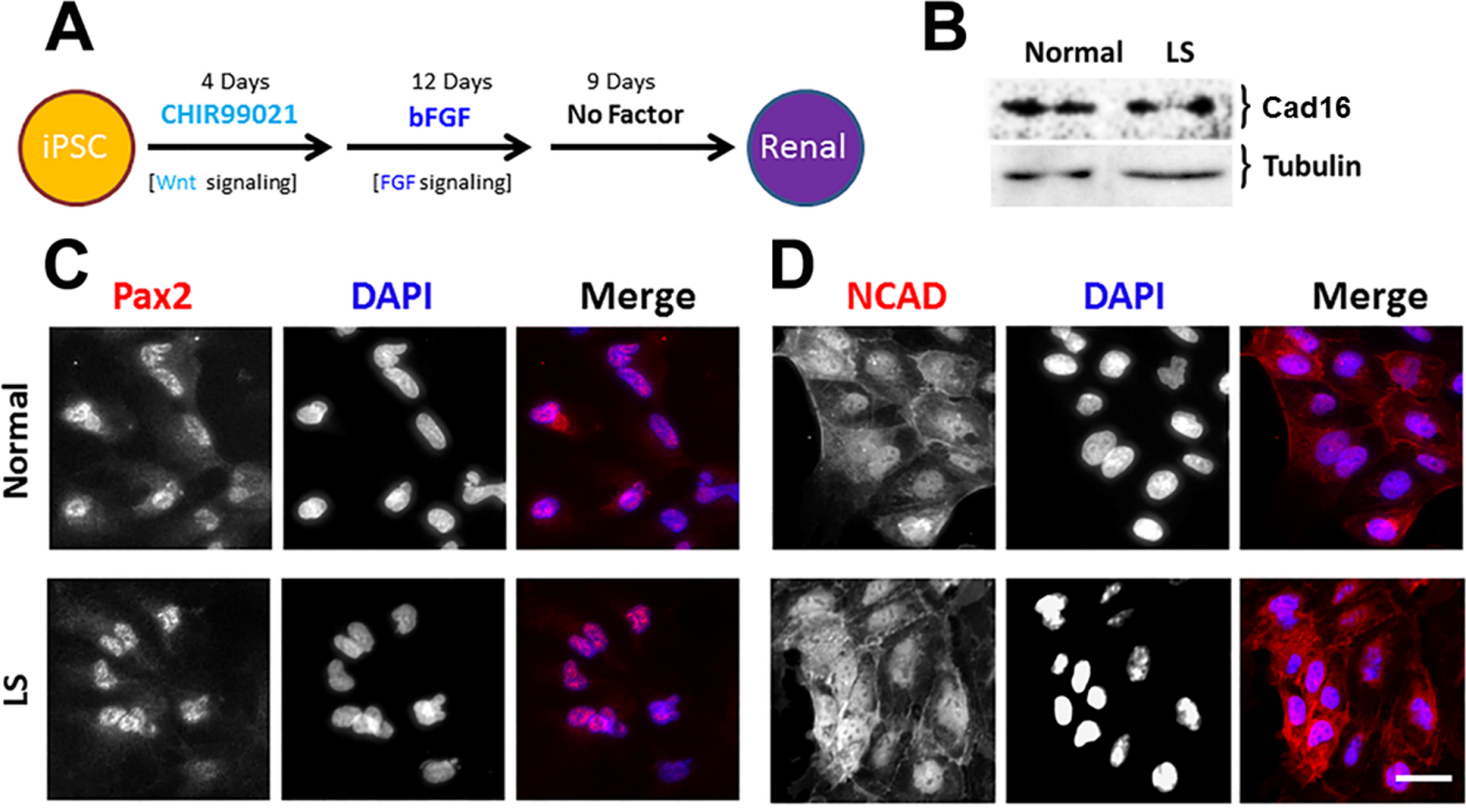
Generation of normal and LS renal cell lineages. **A**. iPSCs were differentiated as renal cells following the procedure described in Materials and methods. **B**. The presence of the kidney-specific marker Cadherin16 (Cad16) was investigated by Western blotting using a specific antibody. Tubulin was detected with a specific antibody and used as loading control. **C** and **D**. Expression and localization of the renal progenitor intermediate mesoderm marker Pax2 and mesendoderm marker N-cadherin (NCAD) was investigated in normal/LS patient differentiated cells using indirect immunofluorescence with specific antibodies (red). DAPI staining (blue) was performed to highlight nucleus position. Scale bar: 10μm.

iPSCs were reprogrammed as kidney cells by following the protocol described in Materials and methods, Fig 2A (and references [37,38]. Briefly, cells were exposed to CHIR99021 (GSK inhibitor) for 4 days, then bFGF was added and maintained until day 17 when it was removed and the cells were kept in the absence of growth factors for 9 additional days. Within that period of time, the presence of renal progenitor markers such as Pax2 and N-Cadherin (intermediate mesoderm and mesendoderm, respectively) and the kidney-specific marker Cadherin16 were verified (Fig 2B–2D). This process was repeated several times out of different iPSC colonies.

#### Kidney-lineage reprogramed LS cells show primary cilia assembly defects

**Fig 3:**
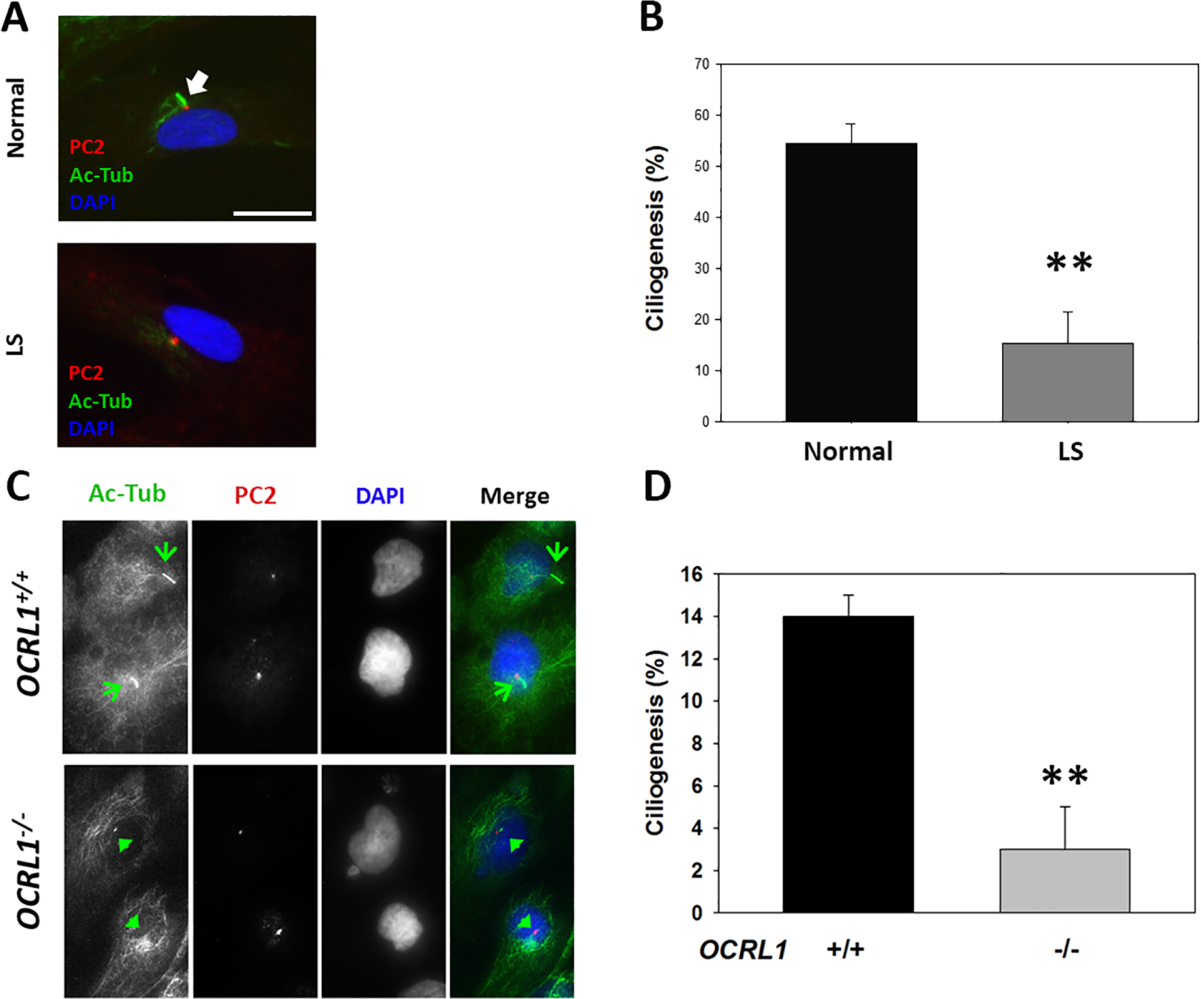
LS renal cells show defects in primary cilia assembly. Ciliogenesis in kidney-differentiated cells was induced as described in Materials and methods. **A** and **C**. Primary cilia (PC) presence was investigated in renal-differentiated (**A**) and HK2 (**C**) cells by indirect immunofluorescence using anti-acetylated tubulin (Ac-Tub, green), anti-pericentrin-2 (PC2, red) antibodies. DAPI staining (blue) was performed to highlight nucleus position. Scale bar: 10μm. **B** and **D**. The percentage of cells displaying PC was determined for normal and LS renal differentiated (**B**) and HK2 (**D**) cells. Statistical significance of difference between means was assessed by using the student *t*-test (**: p<0.05).

We and others have shown that PC assembly is reduced in skin fibroblasts from LS patients and other Ocrl1-deficient cells as compared to normal controls [5–8]. The PC is a specialization of the plasma membrane involved in chemo-, osmo- and mechano-sensing; therefore, it is of high relevance for kidney function [29–32]. In consequence, we proceeded to induce ciliogenesis and to quantify the fraction of kidney-differentiated cells displaying PC (see Materials and methods and [5]). Briefly, PC assembly was induced by serum-starvation and then the cells were fixed and immunostained with an anti-acetylated tubulin antibody to reveal the axoneme and with an anti-pericentrin 2 antibody to label the base of the cilia. The cells were imaged and quantitatively analyzed for PC presence (Fig 3). The data show that LS kidney-reprogrammed cells were impaired for PC assembly as compared to normal controls (Fig 3A and 3B). These results support the idea that PC abnormalities can also manifest in patient renal cells contributing to LS kidney phenotypes and symptoms. We repeated these experiments using differentiated cells from different iPSC colonies. These findings were further verified using a human proximal tubule cell line (HK2) WT and *OCRL1* knockout (see Materials and methods and S1 Fig). Specifically, HK2 *OCRL1* KO cells also displayed ciliogenesis defects as compared to +/+ cells (Fig 3C and 3D). Since this cell line cannot reproduce patient genetic variability, it cannot replace patient’s kidney-reprogrammed cells. However, they had a confirmatory value for the existence of PC phenotypes in kidney cells.

### Cap mesenchyme and nephric duct kidney lineages show disproportionate representation in LS versus normal renal cells

In contrast to differentiation approaches aimed to produce specific cell lineages [39–41], our differentiation strategy was intended to produce several kidney lineages to evaluate the yields of different cell types in LS vs normal cells. Although this strategy highlighted significant differences in the capacity of patient and normal iPSCs to generate renal lineages (see below), it should be kept in mind that *in vivo* differentiation conditions differ and can affect cell type yield.

We monitored two specific kidney lineages: nephric duct and cap mesenchyme, that among others give rise to ductal and tubular cells, respectively [42]. Specifically, we used indirect immunofluorescence to investigate the expression of the Ck8 (ureteric bud) and Six2 (cap mesenchyme) markers for nephric duct and metanephric mesenchyme lineages, respectively [40,43,44]. As expected, a mix of cells positive for Ck8 or for Six2 (it should be noted that expression of Six2 is known to repress ductal genes—[45] was obtained. A fraction of double-negative cells, probably representing other lineages (e.g., stromal or other duct progenitors) was also observed.

**Fig 4:**
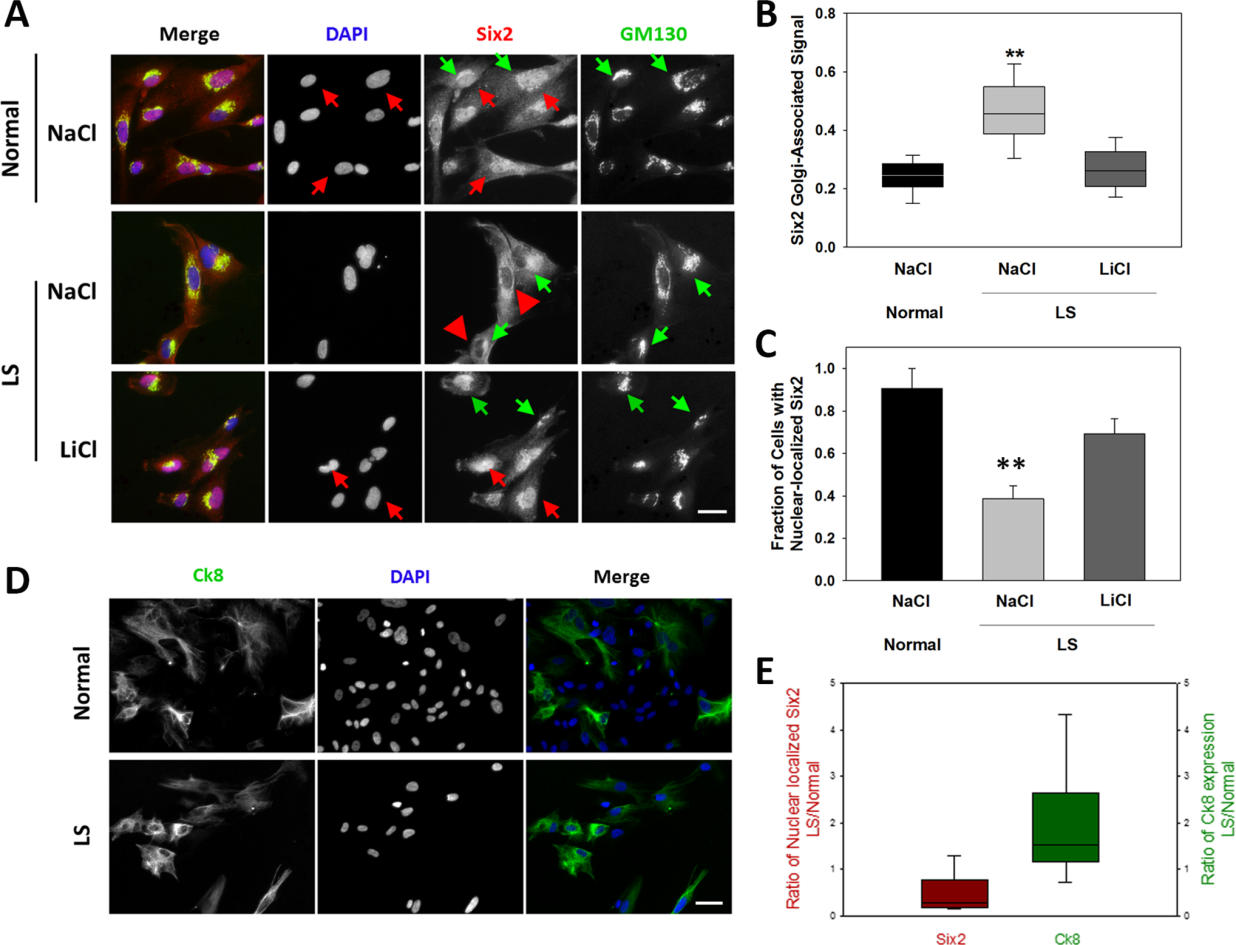
The ductal lineage-inhibitor and cap mesenchyme marker Six2 is retained in the Golgi complex of LS cells. **A**. Normal (upper panels) and LS (middle and lower panels) kidney-differentiated cells treated with NaCl or LiCl were immunostained using anti-Six2 (red) and anti-GM130 (green) antibodies, the location of the nucleus was revealed by DAPI staining (see Materials and methods). Arrows point to some examples of Six2-GM130 colocalization; arrowheads highlight examples of Six2 nuclear exclusion. Scale bar: 10μm. **B-C**. The fluorescence intensity of the Six2-signal associated with the Golgi complex (**B**) or the nucleus (**C**) was quantified and expressed as a fraction of the total Six2 fluorescence (B) or of the number of cells (C) in normal and LS kidney-differentiated cells treated with NaCl or LiCl as indicated. More than 50 cells from at least 5 independent determinations. Statistical significance of the difference between normal and LS samples was assessed using the Wilcoxon test (B) or the *t*-test (C). **: p<0.05. **D**. Representative images of Ck8-positive kidney-differentiated normal and LS cells. Note the increased Ck8^+^ fraction (total Ck8 positive cells/total cells) within LS kidney cells. **E**. Quantification of the relative fraction of LS to normal cells showing Six2 nuclear staining (left in red) and Ck8-positive cells (right in green) in the absence of NaCl or LiCl. In all cases, experiments were repeated using at least 5 differentiation batches (out of different iPSC clones).

Within the Six2-positive population of both normal and LS cells, most showed presence of the Six2 protein in a perinuclear compartment identified as the Golgi complex based on colocalization with the GM130 marker (Fig 4A). Although various intracellular compartments (e.g., lysosomes, endoplasmic reticulum, Golgi apparatus) have been previously shown to act as reservoirs for different transcription factors [46–53], to the best of our knowledge this has not been shown previously for Six2. At least with respect to the direct regulation of gene expression, this Golgi-retained fraction represents an inactive pool of the transcription factor. Interestingly, LS cells showed a significantly higher proportion of Six2 retained at the Golgi complex compared to controls in the presence (Fig 4B) or absence of NaCl (data not shown). Note that NaCl was used as control for LiCl treatment as described below.

In addition, the fraction of cells showing nuclear-localized Six2 was significantly smaller in LS as compared to normal kidney-differentiated cells (Fig 4C and 4E), suggesting an underrepresentation of cap mesenchyme-derived lineages. In contrast, and in agreement with Six2 having an inhibitory role on ductal differentiation [45], Ck8-positive cells were significantly more abundant among LS than normal kidney-differentiated cells (Fig 4D and 4E). Nevertheless, total mRNA and protein levels of Six2 were similar in LS and normal cells based on quantitative RT-PCR, Western blotting and indirect immunofluorescence (S2 Fig). These results were confirmed using different differentiated cell batches produced out of multiple iPSC colonies.

**Fig 5:**
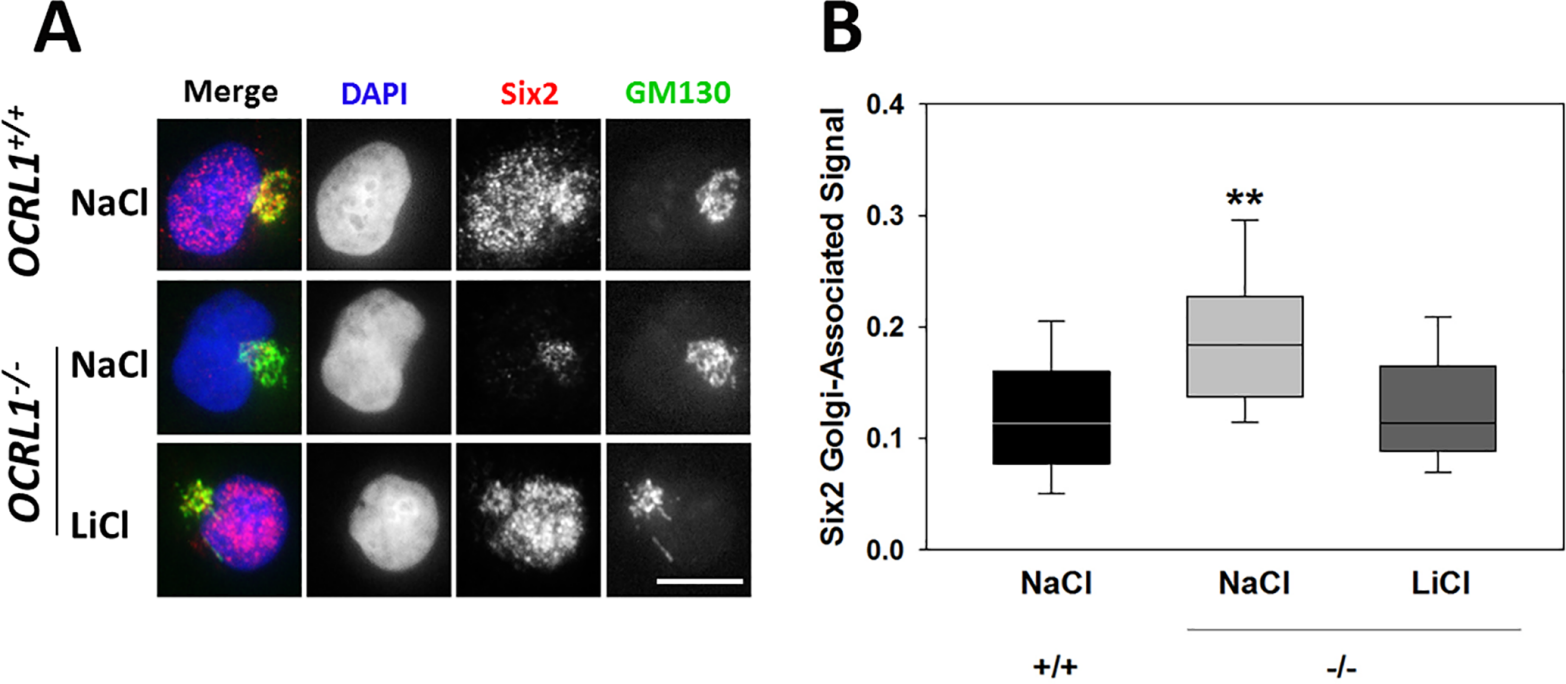
HK2 *OCRL1*^-/-^ proximal tubule cells show Six2 retention in the Golgi complex. WT and *OCRL1* KO (-/-) cells were monitored for Six2 intracellular localization as described in Materials and methods as well as in Fig 4A and 4B. Statistical significance of difference between means was assessed by using the Wilcoxon test (**: p<0.05).

We also analyzed Six2 Golgi-retention in HK2^-/-^ and WT controls. Nevertheless, it should be highlighted that in contrast to iPSC-derived kidney lineages, HK2 cells are *a priori* committed to a cap mesenchyme-derived cells that under Ocrl1-elimination conditions are unlikely to revert to an undifferentiated state or to a different lineage. However, we observed a clear intracellular re-distribution of Six2; specifically, we determined that Six2 was enriched at the Golgi complex in *OCRL1* K.O. in comparison to WT cells (Fig 5).

Interestingly, treatment with 10mM LiCl (a known inhibitor of PI(4,5)P_2_ synthesis—[54,55], counteracted Six2-Golgi retention (Fig 4A and 4B and Fig 5).

## Discussion

LS is a devastating genetic disease that despite being described more than 60 years ago, still lacks a clear delineation of its pathomechanism and no specific cure is available. Among multiple factors contributing to such a scenario, lack of understanding of developmental abnormalities in place and uneven availability of patient cells from affected organs are likely some of them. Here we describe the derivation of iPSCs from LS patient skin fibroblasts and their reprogramming as kidney cells. These kidney-differentiated cells displayed a PC assembly defect, opening the possibility that this phenotype contributes to observed LS renal abnormalities. Interestingly, we also found that in LS cells the balance between ductal and cap mesenchyme kidney lineages is skewed toward the former. In addition, we observed a significant decrease in the fraction of LS cells displaying nuclear localization of the transcription factor Six2, a cap mesenchyme marker that acts as repressor of ductal lineage differentiation. In fact, LS cells displayed a significantly increased pool of Six2 localized at the Golgi complex as compared to normal cells.

The implications of this work are multi-fold; on the one hand, considering the role that the PC plays in osmo/chemo/mechano-sensing, epithelia repair and signaling in general, ciliogenesis abnormalities are expected to affect renal function. Further, we speculate that principles and therapeutic strategies currently attempted against ciliopathies could be conceivably adapted or adopted for treatment of LS.

On the other hand, this work constitutes the first application of iPSC/reprogramming technology to LS. Therefore, it opens the possibility of generating cell types difficult to obtain directly from patients. Specifically, it permits accessible cell types like skin fibroblasts to be converted into cells representative of affected organs, thus providing relevant *in vitro* disease models. Further, these developments will facilitate the preparation of similar samples from multiple patients to better study the effect of different mutations and genetic backgrounds (genetic modifiers) on LS phenotypes. We also anticipate that this work would set up the basis for more sophisticated disease models, such as *in vitro*-generated organoids, and for future cell replacement therapies.

In addition, as exemplified by the present work, iPSC differentiation may provide clues as to how patient embryonic stem cells (ESCs) or adult pools of replenishing cells are affected by the disease. Of course, it should be always kept in mind that iPSCs are not ESCs and that *in vitro* differentiation conditions are not identical to the *in vivo* environment.

Our results showed that kidney-differentiated LS cells exhibited deficient nuclear localization of the transcription factor Six2. In absence of efficient Six2-mediated inhibition, the ductal lineage was overrepresented within the kidney cell population. Multiple implications arise from these observations: First, *within LS kidney cells*, *Six2-dependent cap mesenchyme lineage cells are less readily available*. This observation supports the possibility that LS patients could experience nephron developmental abnormalities, particularly affecting cap mesenchyme-derived cells such as tubular cells. Indeed, it is well-known that LS patients display proximal tubule dysfunction [1,3,56] and tubular atrophy [57]. Indeed, Six2-deficient function has been linked to renal hypoplasia [58,59]. Further, since Six2 is also involved in craniofacial and eye development [59–62] and the protection of dopaminergic neurons [63], it is possible that affected function or regulation of this protein in other tissues or organs leads to other characteristic phenotypes and symptoms of LS.

Second, *patients may have difficulties to replenish cells belonging to the cap mesenchyme lineage following wear*, *injury or various insults*. In fact, there are reports of progressive tubular cell function loss in LS patients [57]. Although the origin/nature of renewable stem cell-like cells in the post-natal kidney is still controversial [64–66], there is a body of evidence supporting their existence and relevance for maintaining renal functionality [66–68]. Our work suggests the availability of such cell replenishing pool may be compromised in LS patients.

Third, *our findings provide the first observation of the Golgi complex playing a role in Six2 intracellular distribution and function*. In addition, these findings constitute another example of the Golgi apparatus acting as a reservoir or sink for a specific transcription factor [47,48]. It should be noted that Six2 localization at the Golgi apparatus was also detected in normal cells, suggesting that this organelle may play a physiological role in regulating Six2 transcription factor activity by serving as a reservoir to control the size of the nuclear translocation-competent pool. Alternatively, the absence of Ocrl1 (a Golgi complex-localized protein) may lead to changes (such as differences in protein/lipid composition) that yields an abnormal enrichment of Six2 in this organelle, something of only minor incidence in normal cells.

The mechanism by which Six2 is mislocalized in LS renal cells and whether this depends on the accumulation of PI(4, 5)P_2_ (known to take place in the absence of functional Ocrl1) or the absence of critical protein complexes involving Ocrl1, will be the focus of future investigations. It is interesting to note that treatment with LiCl is known to lower PI(4, 5)P_2_ levels [54,55] and to also enhance Six2 functional activity [69]. Indeed, incubation with LiCl decreased the Golgi localization of Six2 in both LS renal cells and HK2 *OCRL1* K.O. cells. However, it is uncertain if this effect is the result of counteracting PI(4, 5)P_2_ accumulation at the Golgi apparatus, endosomes, cilia [70] or all. Further, it is conceivable that accumulation of PI(4,5)P_2_ contributes to both ciliogenesis abnormalities [71] and Six2-retention at the Golgi apparatus. However, the mechanism underneath the cilia assembly defects is expected to also involve vesicle trafficking abnormalities [5] and a role for Six2 altered function cannot be discarded and should be the object of future investigations. In addition, the occurrence of Six2 sequestration at the Golgi complex should be confirmed and eventually studied in LS animal models to better understand its potential contribution to the disease.

## Materials and methods

### Reagents

Materials were purchased from Fisher Scientific (Fairlawn, NJ) or Sigma (St. Louis, MO) unless stated otherwise. The different antibodies used in this study are listed inS1 Table.

### Cells and culture conditions

Normal (GM07492) and LS primary dermal fibroblasts (GM 03265) were obtained from the NIHGMS Human Genetic Cell Repository (Coriell Institute for Medical Research, Camden, NJ, USA). Cells were maintained in DMEM, Streptomycin/Penicillin, 2mM L-Glutamine and 15% fetal bovine serum (FBS) at 37°C in a 5% CO2 incubator.

HK2 *OCRL1* knockout cell line was prepared by using the CRISPR/Cas9 system. Isogenic *OCRL1* knockout HK2 cell clones were generated by diluting and cultivating the transfected cells in 96-well plates and were identified by Sanger sequencing. Western blotting of lysates prepared from the knockout cells confirmed absence of Ocrl1 protein (S1 Fig).

### Western blotting

Cell lysates in Laemmli's protein sample buffer were separated on 10% gels at 40mA constant current in SDS/PAGE running buffer (100mM Tris base, 100mM Hepes, 0.1% SDS) and transferred onto nitrocellulose membrane in transfer buffer (48mM Tris, 1mM SDS, 400mM glycine,10% methanol) at 80V for 2h. Blots were blocked 1h at room temperature in PBST-milk (137mM NaCl, 10mM Na_2_HPO_4_, 2.7mM KCl, 0.1% Tween-20, pH = 7.4, 5% non-fat dried milk) and incubated with the appropriate primary antibody and dilution (anti-Ocrl1:150; Santa Cruz- sc-393577, anti-tubulin: 1:500; Biolegend- 627903) overnight at 4°C or for 1h at room temperature respectively. After incubation with a secondary antibody conjugated with horseradish peroxidase for 1h at room temperature and washing, specific bands were detected by chemiluminescence using SuperSignal West Femto (Pierce) as a substrate and visualized using an Alpha-Innotech imaging system (San Leandro, CA, USA).

### Preparation of iPSC from human skin fibroblast

Human normal and LS skin fibroblasts were plated at a density of 10^5^ cells/well in 6-well plates and maintained at 37°C, 5% CO_2_ for 6h. The cells were infected witha retrovirus cocktail at MOI 17.5 for expression of hOCT4, hSOX2, hKLF4 and hc-MYC genes (ALSTEM) and 4μl of 500x TransPlus (ALSTEM) in 2ml of fresh DMEM medium containing 15% FBS. The infection was repeated on the next day. On the 5^th^ day after first infection, the cells were trypsinized and seeded on a 60mm plate coated with 0.1% gelatin containing 10^6^ mitomycin-c treated, mouse embryonic fibroblasts (MEF). On the 7^th^ day, the culture medium was replaced by iPSC medium (DMEM/F12, 20% Knock-out serum replacement, 1% Penicillin/Streptomycin, 1% Non-essential amino-acids, 0.1mM Beta-mercapto-ethanol, 10ng/ml bFGF (StemRD)). The iPSC medium was replaced daily for about 5 weeks until colonies appeared. The cells were live-stained with an anti Tra-1-60 antibody conjugated with DyLight™ 488 (Stemgent) and positive colonies identified by epifluorescence microscopy using a GFP filter. The pluripotency of the cells was further verified by immunofluorescence and qPCR. iPSC cells were cultured on feeder layers of mitomycin-c treated C57Bl/6 MEF with daily replacement of fresh iPSC medium. The cells were passaged weekly at a ratio of 1:3–1:5.

*Karyotyping*: Actively dividing iPSC were treated with 0.1μg/ml Demecolcine (MP Biomedicals) for 3h at 37°C. The colonies were dissociated into single cells, and incubated with hypotonic solution (KCl 75mM) for 20min in 37°C and fixed with methanol/glacial acetic acid 3:1, overnight. Cells were washed thrice with fixation solution and were dropped on to slides and dried on a 95°C hot-plate. The samples were stained with 2% Giemsa solution (Eelectron microscopy sciences), and imaged at 100x.

### Indirect immunofluorescence

All cells were grown on coverslips and fixed with 4% formaldehyde/PBS for 10min at room temperature. After washing with PBS, the iPSC cells were incubated with the corresponding primary antibody diluted in blocking solution (10% FBS containing DMEM medium with 0.1% saponin) at 4°C, overnight while HK2 cells (WT and *OCRL1* KO) were stained with specific primary antibodies (see S1 Table) for 1h at room temperature, Following two washes with PBS, the cells were incubated with appropriate secondary antibodies diluted in blocking solution for 45min at room temperature. The cells were washed and stained with DAPI for 5min, washed and mounted on slides with Aqua-PolyMount (Polysciences).

Images were acquired using a Zeiss Axioimager.Z1 microscope equipped with Zeiss Axiocam MRm monochrome digital camera and Carl Zeiss Axiovision image acquisition software.

### Quantitative RT-PCR

Total RNA was collected from cells by using Direct-zol kit (Zymo research) following manufacturer’s protocol. 100ng of total RNA for each sample were used for cDNA synthesis and qRT-PCR. The assays were performed using the qScript One-Step SYBR Green RT-qPCR kit (Quanta bio) in a Roche LightCycler 96 real-time PCR machine. The target mRNA expression levels in iPSC were normalized with respect to RPLP0 messenger levels and expressed as fraction of the fibroblast values using the ΔΔCq method. The sequences of the primers used are listed in S2 Table.

### Differentiation of iPSC into kidney cells

iPSC colonies were lifted from the feeder layers by incubation with detaching solution (1mM CaCl_2_, 2mg/ml Collagenase IV, 0.125% trypsin in DMEM) at 37°C until the edges of the colonies were lose. The colonies were collected by scratching them with a p200 tip and transferred to 0.1% gelatin coated plates for 1h at 37°C, 5% CO_2_ in iPSC medium to remove the MEF cells. The colonies were collected into microcentrifuge tubes and pelleted at 300xg for 5min, washed once with PBS, and incubated with Accutase (Innovative cell technologies) for 30min at 37°C and gently dissociated into single cells by flicking the microcentrifuge tubes. The cells were washed once in PBS and resuspended in basal medium (DMEM/F12, 20% Knock-out serum replacement, 1% Penicillin/Streptomycin, 1% Non-essential amino-acids, 0.1mM Beta-mercapto-ethanol) with 5μM CHIR99021(Biovision) and 10μM Y27632(LC Laboratories). The cells were seeded onto wells coated with 5% growth factor-reduced matrigel (BD) at density of 1.5x10^4^ cells/cm^2^. The following 3 days (days 2–4) the cells were treated with 8μM CHIR99021 in basal medium. On day 5, the cells were treated with 200ng/ml bFGF in basal medium, the medium was replaced every other day for 12 days (until day 17). On day 18, bFGF was removed from the culture medium; and the medium was replaced every other day for 9 additional days; after that period, the differentiated cells were used. The process was repeated at least 5 times out of several normal and LS iPSC colonies.

### Ciliogenesis

The renal cells were seeded onto 22x22 mm^2^ coverslips at 60% confluency in 15% FBS containing DMEM medium for 12h followed by serum-starvation by incubating them in 0.1% serum medium for 48h and by 24h at 0% serum medium. HK2 cells (WT and *OCRL1* KO) were plated on coverslips in complete media (containing 10% FBS) and allowed to attach overnight to achieve ~80% confluency and starved for 48h incubation with media containing 1% FBS.

The cells were fixed in 4% formaldehyde for 10min at room temperature. Then the samples were immunostained with anti-acetylated tubulin and anti-pericentrin-2 primary antibodies followed by addition of appropriate secondary antibodies. The nucleus of the cells was stained with DAPI. Level of ciliogenesis was determined by imaging the cells using fluorescence microscopy followed by determination of the percentage of total cells that has an acetylated tubulin marked primary cilium emerging from a pericentrin-positive structure. More than 150 cells were analyzed per sample; two-tailed *t*-test was performed for statistical analysis.

## Supporting information

S1 Table Antibodies used in this study. (PDF)

S2 Table Primers used in this study. (PDF)

S1 Fig Ocrl1 levels in fibroblasts and HK2 cells. Lysates from Normal and LS patient fibroblasts as well as HK2 WT (+/+) and *OCRL1* KO (-/-) were resolved by SDS-PAGE and the presence of Ocrl1 was investigated by Western blotting using a specific antibody. Tubulin was detected with a specific antibody and used as loading control.
(TIF)

S2 Fig The transcription factor Six2 is equally expressed in LS and normal kidney-differentiated cells. Results from quantitative RT-PCR (qRT-PCR), Western blotting and quantitative Indirect Immunofluorescence (qIIF) are shown. Left panel shows normal to LS relative ratio quantifications of Six2 expression levels from at least 3 independent experiments. Right upper and lower panels show representative Six2 detection results using immunofluorescence and Western blotting, respectively. Scale bar: 20μm.
(TIF)
