# Supplementary material for: Kidney-differentiated cells derived from Lowe Syndrome patient’s iPSCs show ciliogenesis defects and Six2 retention at the Golgi complex
Source: PLoS One. 2018 Feb 14;13(2):e0192635. doi: 10.1371/journal.pone.0192635 (PMC5812626; doi:10.1371/journal.pone.0192635)
Supplement: S1 Table — (PDF) [file pone.0192635.s001.pdf]

Supplemental Table I: Antibodies used in this study				
**Antibody**	**Source**	**Cat#**	**Host**	**Dilution**
Anti-Tra-1-60	Stemgent	09-0068	Mouse	1:100
Anti-OCT-4A	Cell signaling	2840	Rabbit	1:400
Anti-E-CAD	Abcam	Ab1416	Mouse	1:100
Anti-N-CAD	Abcam	Ab18203	Rabbit	1:400
Anti-PAX2	Genetex	GTX54602	Rabbit	1:2000
Anti-Tubulin (Acetyl Lys40)	Genetex	GTX16292	Mouse	1:1000
Anti-Pericentrin 2	Millipore	ABT59	Rabbit	1:500
Anti-GM130	BD bioscience	610822	Mouse	1:75 (differentiated renal cells)<br>1:200 (HK2 cells)
Anti-SIX2	Proteintech	11562-1-AP	Rabbit	1:200(IF)<br>1:600 (WB)
Anti-Cytokeratin 8	Abcam	Ab9023	Mouse	1:200
Anti-Cad16	Abcam	Ab80320	Rabbit	1:500
Anti-Rabbit IgG-Cy3	Jackson immunoresearch	111-165-144	Goat	1:400
Anti-Mouse IgG-Cy2	Jackson immunoresearch	111-165-146	Goat	1:400
