# Supplementary material for: Kidney-differentiated cells derived from Lowe Syndrome patient’s iPSCs show ciliogenesis defects and Six2 retention at the Golgi complex
Source: PLoS One. 2018 Feb 14;13(2):e0192635. doi: 10.1371/journal.pone.0192635 (PMC5812626; doi:10.1371/journal.pone.0192635)
Supplement: S2 Table — (PDF) [file pone.0192635.s002.pdf]

Supplemental Table II: Primers used in this study	
**Gene**	**Primer sequence**
OCT3/4	GACAGGGGGAGGGGAGGAGCTAGG
	CTTCCCTCCAACCAGTTGCCCCAAAC
SOX2	GGGAAATGGGAGGGGTGCAAAAGAGG
	TTGCGTGAGTGTGGATGGGATTGGTG
Nanog	TCTCTCCTCTTCCTTCCTCCATG
	CTGTTTGTAGCTGAGGTTCAGGATG
DNMT3B	TGCTGCTCACAGGGCCCGATACTTC
	TCCTTTCGAGCTCAGTGCACCACAAAAC
FGF4	CTACAACGCCTACGAGTCCTACA
	GTTGCACCAGAAAAGTCAGAGTTG
SIX2	AGGAAAGGGAGAACAACGAGAA
	GGGCTGGATGATGAGTGGT
RPLP0	CGCAGCCAATAGACAGGAG
	GCGCGTGCCTTTTATAATGC
