# Supplementary figures and images for: Kidney-differentiated cells derived from Lowe Syndrome patient’s iPSCs show ciliogenesis defects and Six2 retention at the Golgi complex
Source: PLoS One. 2018 Feb 14;13(2):e0192635. doi: 10.1371/journal.pone.0192635 (PMC5812626; doi:10.1371/journal.pone.0192635)

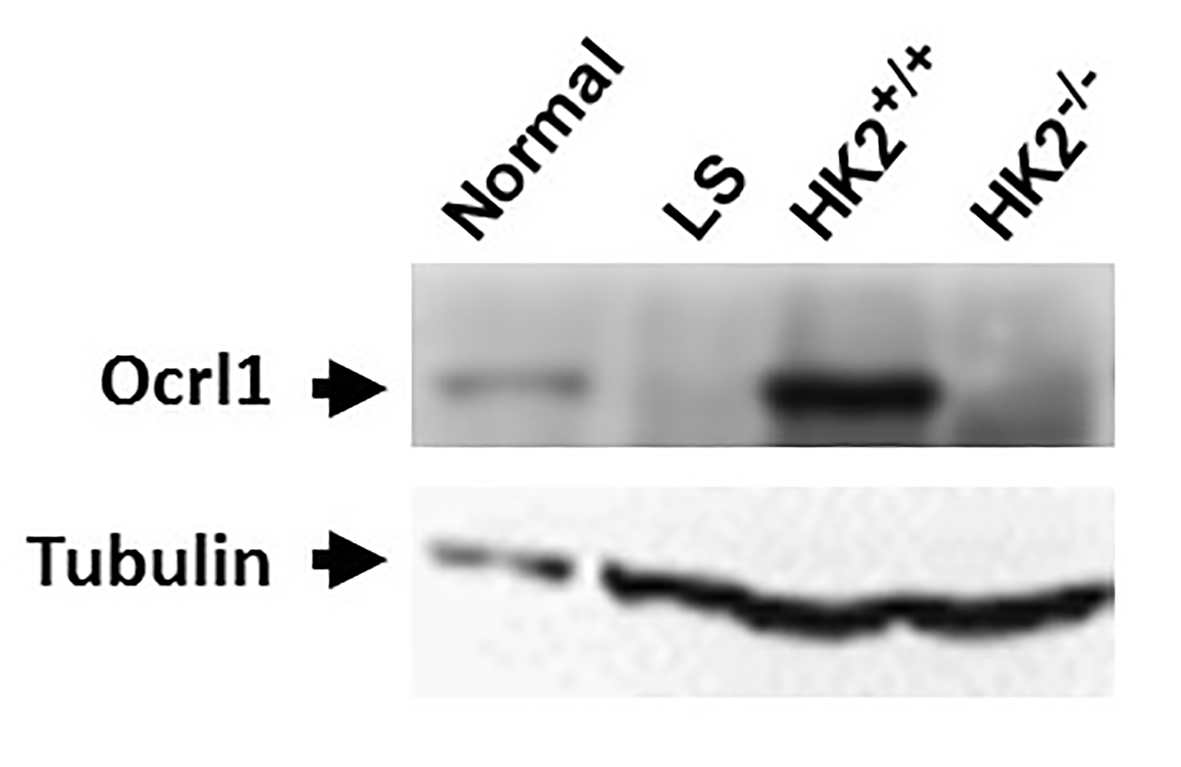

Supplement: S1 Fig — Lysates from Normal and LS patient fibroblasts as well as HK2 WT (+/+) and OCRL1 KO (-/-) were resolved by SDS-PAGE and the presence of Ocrl1 was investigated by Western blotting using a specific antibody. Tubulin was detected with a specific antibody and used as loading control. (TIF) [file pone.0192635.s003.tif]

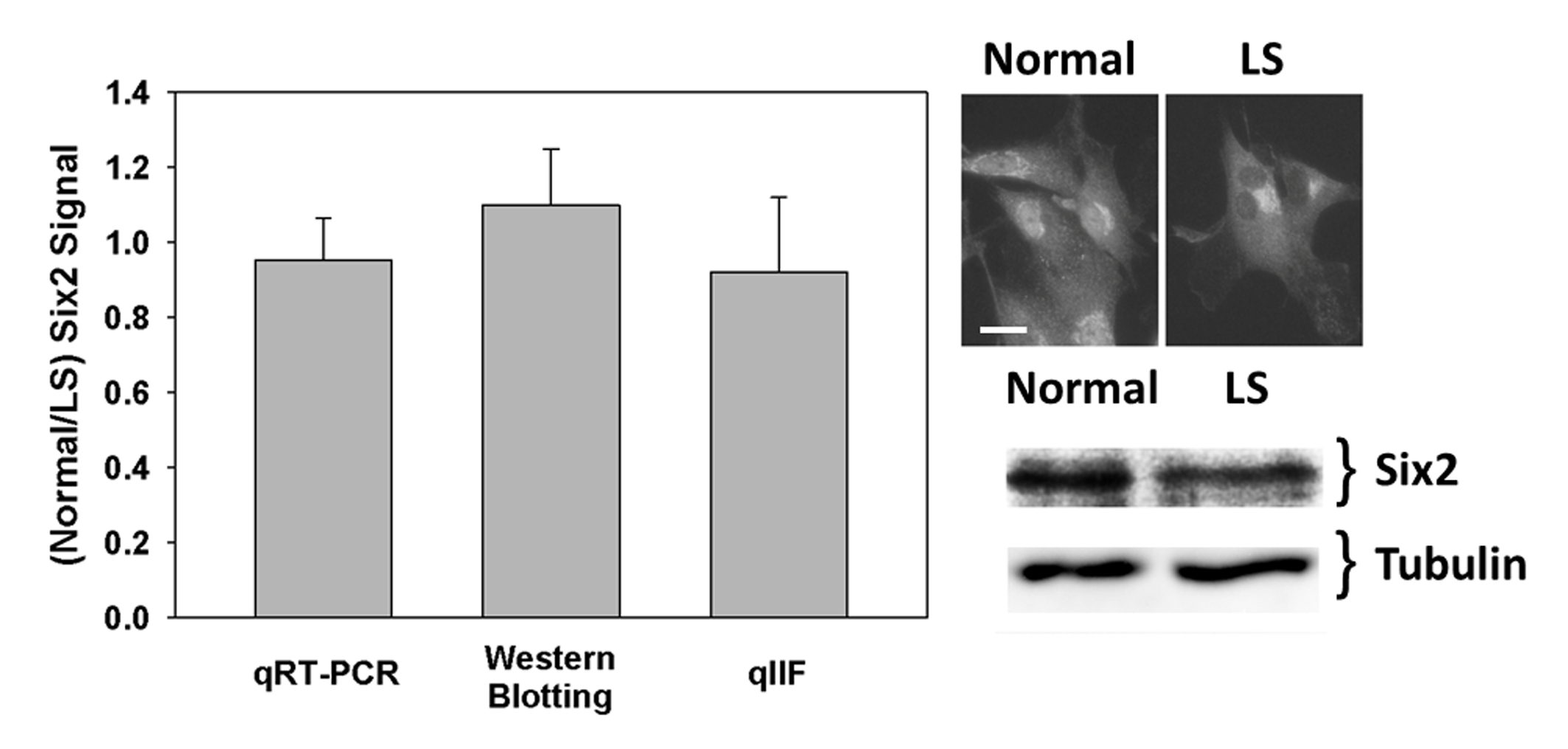

Supplement: S2 Fig — Results from quantitative RT-PCR (qRT-PCR), Western blotting and quantitative Indirect Immunofluorescence (qIIF) are shown. Left panel shows normal to LS relative ratio quantifications of Six2 expression levels from at least 3 independent experiments. Right upper and lower panels show representative Six2 detection results using immunofluorescence and Western blotting, respectively. Scale bar: 20μm. (TIF) [file pone.0192635.s004.tif]
